# Draft Genome Sequences of Six Novel Picorna-Like Viruses from Washington State Spiders

**DOI:** 10.1128/genomeA.01705-16

**Published:** 2017-03-02

**Authors:** Ryan C. Shean, Negar Makhsous, Rodney L. Crawford, Keith R. Jerome, Alexander L. Greninger

**Affiliations:** a Department of Laboratory Medicine, University of Washington, Seattle, Washington, USA; b Burke Museum, University of Washington, Seattle, Washington, USA; c Vaccine and Infectious Disease Division, Fred Hutchinson Cancer Research Center, Seattle, Washington, USA

## Abstract

We report draft genome sequences of six novel *Picornavirales* members from six different spider species found in Washington state. These six viral sequences distinctly clustered together phylogenetically with less than 35% amino acid identity to the closest reference viral genome.

## GENOME ANNOUNCEMENT

As new arthropod-borne zoonoses become of increasing relevance to human health it has become clear that greater understanding of the arthropod virome is required (1). Spider metagenomics provides an ample area for viral discovery, as spiders both are undersequenced and act as arthropod aggregators that are windows into the greater invertebrate virosphere (2).

All spiders were sampled in western Washington and stored in ethanol for <1 year before sequencing. *Hexura picea* Simon is a native mygalomorph spider of the family *Mecicobothriidae*; *Metellina curtisi* (McCook) is a native orbweaver of the family *Tetragnathidae*; *Hyptiotes gertschi* Chamberlin & Ivie is a native triangle-weaving orbweaver. *Theridion simile* C. L. Koch is a native cobweb weaver of Holarctic distribution. *Theridion tinctum* (Walckenaer) is an introduced cobweb weaver and *Xysticus cristatus* (Clerck) is an introduced crab spider, both of European origin.

Metagenomic libraries were performed as described previously (3). Lysates from bead-beaten spiders were spin-filtered through a 0.45 µM filter and RNA was extracted. Double-stranded cDNA was tagmented using Nextera XT and amplified with 19 cycles of PCR (4). Adapter-trimmed reads were assembled SPAdes v3.8 and translated open reading frames (ORFs) >2 kb were aligned to PDB via HHPred (5, 6).

From the *Theridion simile* spider, an 8,825 nucleotide (nt) contig with one continuous open reading frame was assembled corresponding to the novel *Picornavirales* Rainier virus UW1. The translated open reading frame aligned 29% by amino acid to *Solenopsis invicta* virus-1 (NC_006559) (7). From the *Xysticus cristatus* spider, a 10,001 nucleotide contig containing a singular ORF was assembled, corresponding to Crab spider picornavirus. The translated ORF aligned 28% by amino acid to the Israeli acute paralysis virus (8).

From the *Hexura picea* spiders, two contigs of 9,798 and 10,025 nt were assembled with ORFs of 9,734 and 9,922 nt, respectively. The translated ORFs aligned 82% by amino acid, constituting two strains (BG1 and RC1) of the novel Burke-Gilman virus. They aligned 33% by amino acid to the polyprotein of the Rice tungro spherical virus, a member of the *Secoviridae* family (9). These two viruses also aligned 31 to 33% by amino acid to the Rainier virus UW1, Crab spider picornavirus, and Baker virus.

Partial genome sequences from three novel species from this likely novel clade were also recovered. Metagenomic sequences from a *Metellina curtisi* spider contained a 3,992 nt contig that aligned most closely to the aphid lethal paralysis virus with an amino acid identity of 24%. This contig corresponded to the structural protein of Volivirus B7-MC0. The *Hyptiotes gertschi* assembly contained a contig of 3,256 bases (Swagivirus HG) and aligned 35% by amino acid to the helicase of Aichi virus 1 (10). The *Theridion tinctum* assembly included a contig of 2,421 bases and aligned 47% by amino acid to the Aphis glycines virus 1 capsid polyprotein (KF360262).

These viruses likely form a new clade within the *Picornavirales* order as they cluster closer to each other than to any of the reference viral sequences listed here. Further taxonomic, sequencing, and functional studies would be required to fully delineate their place within the *Picornavirales* order.

### Accession number(s).

The sequences described in this study are deposited in DDBJ/ENA/Genbank under the accession numbers KX779450 (Crab spider picornavirus J1), KX782311 and KX782312 (Burke-Gilman virus RC1 and BG1), KX782313 (Rainier virus UW1), KY316057 (Noveria virus B8-Tt), KY316058 (Volivirus B7-MC0), and KY316059 (Swagivirus HG B7-Hg).

## References

[1] Coffey LL, Page BL, Greninger AL, Herring BL, Russell RC, Doggett SL, Haniotis J, Wang C, Deng X, Delwart EL 2014 Enhanced arbovirus surveillance with deep sequencing: identification of novel rhabdoviruses and bunyaviruses in Australian mosquitoes. Virology 448:146–158. doi:10.1016/j.virol.2013.09.026. 24314645 PMC3870893

[2] Shi M, Lin XD, Tian JH, Chen LJ, Chen X, Li CX, Qin XC, Li J, Cao JP, Eden JS, Buchmann J, Wang W, Xu J, Holmes EC, Zhang YZ 2016 Redefining the invertebrate RNA virosphere. Nature 540:539–543. doi:10.1038/nature20167. 27880757

[3] Greninger AL, Naccache SN, Messacar K, Clayton A, Yu G, Somasekar S, Federman S, Stryke D, Anderson C, Yagi S, Messenger S, Wadford D, Xia D, Watt JP, Van Haren K, Dominguez SR, Glaser C, Aldrovandi G, Chiu CY 2015 A novel outbreak enterovirus D68 strain associated with acute flaccid myelitis cases in the USA (2012-14): a retrospective cohort study. Lancet Infect Dis 15:671–682. doi:10.1016/S1473-3099(15)70093-9. 25837569 PMC6027625

[4] Greninger AL, Zerr DM, Qin X, Adler AL, Sampoleo R, Kuypers JM, Englund JA, Jerome KR 2017 Rapid metagenomic next-generation sequencing during an investigation of hospital-acquired human parainfluenza virus 3 infections. J Clin Microbiol 55:177–182. doi:10.1128/JCM.01881-16. 27795347 PMC5228228

[5] Bankevich A, Nurk S, Antipov D, Gurevich AA, Dvorkin M, Kulikov AS, Lesin VM, Nikolenko SI, Pham S, Prjibelski AD, Pyshkin AV, Sirotkin AV, Vyahhi N, Tesler G, Alekseyev MA, Pevzner PA 2012 SPAdes: a new genome assembly algorithm and its applications to single-cell sequencing. J Comput Biol 19:455–477. doi:10.1089/cmb.2012.0021. 22506599 PMC3342519

[6] Hildebrand A, Remmert M, Biegert A, Söding J 2009 Fast and accurate automatic structure prediction with HHpred. Proteins 77:128–132. doi:10.1002/prot.22499. 19626712

[7] Valles SM, Strong CA, Dang PM, Hunter WB, Pereira RM, Oi DH, Shapiro AM, Williams DF 2004 A picorna-like virus from the red imported fire ant, *Solenopsis invicta*: initial discovery, genome sequence, and characterization. Virology 328:151–157. doi:10.1016/j.virol.2004.07.016. 15380366

[8] Maori E, Lavi S, Mozes-Koch R, Gantman Y, Peretz Y, Edelbaum O, Tanne E, Sela I 2007 Isolation and characterization of Israeli acute paralysis virus, a dicistrovirus affecting honeybees in Israel: evidence for diversity due to intra- and inter-species recombination. J Gen Virol 88:3428–3438. doi:10.1099/vir.0.83284-0. 18024913

[9] Shen P, Kaniewska M, Smith C, Beachy RN 1993 Nucleotide sequence and genomic organization of rice tungro spherical virus. Virology 193:621–630. doi:10.1006/viro.1993.1170. 8460478

[10] Yamashita T, Kobayashi S, Sakae K, Nakata S, Chiba S, Ishihara Y, Isomura S 1991 Isolation of cytopathic small round viruses with BS-C-1 cells from patients with gastroenteritis. J Infect Dis 164:954–957. doi:10.1093/infdis/164.5.954. 1658159

